# Extraction of Triterpene Compounds From *Hancornia speciosa* Gomes Fruits and Evaluation of Pharmacological Potential

**DOI:** 10.1002/cbdv.202501034

**Published:** 2025-07-14

**Authors:** Sérgio Prado Leite, Michel Rubens dos Reis Souza, Adilson Allef Moraes Santana, Monalisa Martins Montalvão, Camilla Natália Oliveira Santos, Lucas Sousa Magalhães, Cristiane Bani Correa, Tatiana Rodrigues de Moura, Sona Jain, Thiago Rodrigues Bjerk

**Affiliations:** ^1^ Universidade Tiradentes Aracaju Brazil; ^2^ Universidade Federal de Sergipe Sao Cristovao Brazil

**Keywords:** chromatography, *Hancornia speciosa*, mangaba, medicinal plants

## Abstract

*Hancornia speciosa* Gomes, a Brazilian native species, holds biotechnological potential due to compounds such as lupeol, α‐amyrin, and β‐amyrin, which possess pharmacological properties. This study investigates triterpene‐rich extracts from *H. speciosa* fruit for potential anti‐inflammatory applications. Extraction was performed using the energized‐guided dispersive extraction (EDGE) method and compared to ultrasound extraction techniques to evaluate efficiency and yield. The EDGE system demonstrated notable advantages over ultrasound extraction, including a 60 mL reduction in solvent volume and an 87‐min decrease in extraction time, enhancing efficiency, cost‐effectiveness, and sustainability. The EDGE extraction successfully obtained a triterpene‐rich extract from *H. speciosa* Gomes with a concentration of lupeol (50.09 ± 1.29 mg/g) with no significant difference when compared to the extract obtained by ultrasound (49.95 ± 2.54 mg/g). The extract showed no cytotoxicity and irritation in the hen's egg test‐chorioallantoic membrane assay and significantly reduced reactive oxygen species produced by neutrophils. These results indicate the extract's phytotherapeutic potential, suggesting new possibilities for future studies and therapeutic applications, and highlight EDGE as a promising technique for phytotherapeutic applications.

## Graphical abstract

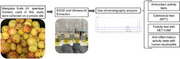

## 1 Introduction

*Hancornia speciosa* Gomes, popularly known as mangabeira, is a medium‐sized arboreal tree, ranging from 4 to 7 m. Its leaves are generally simple, opposite, elliptical, oblong, or elliptical‐lanceolate at both ends. The flowers are white, ellipsoidal or rounded, and their fruiting can occur at different periods of the year, mainly from July to October and from January to April [1, 2, 3].

In addition to Brazil, where it is native, it can also be found in other countries such as Paraguay, Bolivia, and Peru. In Brazil, *H. speciosa* Gomes is found in the coastal plateau, savanna, and plateau, with greater abundance in the coastal lowlands of the Northeast [1, 2, 4]. Recent studies have indicated anti‐inflammatory [5, 6, 7], antihypertensive [8, 9], antioxidant [10, 11, 12, 13, 14], and antidiabetic [15, 16] activity using different parts of the plant.

Among the possible compounds identified in *H. speciosa*, the triterpenes lupeol, α/β‐amyrin and their acetates stand out for their diverse pharmacological activities, from effects against inflammation [17, 18, 19, 20, 21, 22, 23], diabetes [19, 23], cardiovascular diseases [19, 23], microbial infections [19, 20, 23] and tumor [19, 21, 22, 23].

Energized‐guided dispersive extraction (EDGE) system is a modern, automated, and promising technique that combines pressurized liquid extraction and solid‐phase extraction and is considered faster and simpler than other solvent extraction systems [24]. This technique uses a pressurized seal on the container to keep the solvents in liquid form during the extraction process and accelerate penetration into the sample pores. High pressure allows extraction to operate at temperatures above the boiling point of the solvent, forcing it into areas of the sample that would normally be possible using atmospheric conditions [25, 26].

To carry out the extraction process, the sample must be loaded into a container (Q‐Cup), which contains a filter (Q‐Disc) at the bottom of the container, followed by the selection of the extraction method and the number of samples to be extracted. During extraction, the container is automatically loaded with solvents by the sampler and is then pressure sealed at the top. The solvent is first added from the bottom, filling the gap between the camera and the container, helping with heat transfer, and then the solvent is added from the top into the container while the chamber walls are heated (minimum temperature of 25°C and maximum 200°C). Finally, the extracted solvent and analytes are filtered and collected in the collection bottle, ready to be analyzed [24].

Different studies have reported optimized extractions using EDGE with different species of plants, and through the results obtained, it was possible to identify the effectiveness of the method used, especially due to lower solvent consumption, shorter extraction times, and better reproducibility [27, 28, 29].

In view of the reports of the application of *H. speciosa* Gomes in popular medicine and its pharmacological potential, the article aimed to extract lupeol and other triterpenes from the fruits of *H. speciosa*, using the EDGE extraction method, and to explore the potential pharmacological activities.

## 2 Results and Discussion

### 2.1 Extraction With EDGE and Chromatographic Analysis

Initially, analysis of the extracts was carried out at the central point to identify the chromatographic profile of the sample. The gas chromatograph coupled to a mass spectrometry detector with a quadrupole mass analyzer (GC/qMS) chromatograms of the dichloromethane (DCM) and hexane (HEX) extracts showed similar profiles, showing the presence of compounds with similar structural characteristics in both fractions. Figure S1a, available in the Supporting Information, shows the comparison between the chromatograms of the two solvents used.

From the data obtained, it was observed that the compounds with the highest percentage area of the extracts were lupeol acetate (average of 30.04% in the DCM extract and 30.57 in the HEX extract), lupeol (average of 23.33% in DCM extract and 22.63 in HEX extract) and hexadecanal (average of 15.06% in DCM extract and 14.98 in HEX extract). It was noted that, among all the compounds identified in the DCM and HEX extract, triterpenes represent, respectively, 64.22% and 64.34% of the sample area. The analysis of the major triterpenes, lupeol and lupeol acetate, by GC/MS showed a good separation, which allows these compounds to be analyzed quantitatively.

The mangaba fruit was extracted with the solvents DCM and Hexane, using a 2^3^ experimental design. Ultrasound‐assisted extraction was also performed using the conditions indicated in a previous study [30] to compare the concentration of quantified triterpenes between the methods. The extraction conditions were 3 g of sample, 20 mL of ethanol, and 60 mL of DCM, 90 min of extraction, temperature of 30°C.

The chromatograms of the extracts that showed the best results can be seen in Figure S1b. It is possible to observe the similarity of the chromatographic profile obtained between the different methods used.

### 2.2 Quantitative Analysis of Lupeol and Triterpenes

An analytical curve was constructed for the lupeol solution in DCM, at concentrations of 20, 40, 60, 80, 120, and 140 µg/mL, containing 10 µg/mL of the internal standard, using the following equation:

$$y=7.598596e-003x-8.474174e-003\;\mathrm{and}\;R^{2}\;=0.999203$$

The concentration of the triterpene lupeol in the DCM and Hexane samples is shown in Table 1.

**TABLE 1:** Mean lupeol concentration, quantitatively evaluated, present in *H. Speciosa* Gomes extracts obtained by energized‐guided dispersive extraction (EDGE). The highest concentrations of lupeol obtained in each solvent used are indicated in the table.

Experiment	Mean DCM	Mean HEX
**Exp 1**	40.23 ± 0.58	41.63 ± 1.95
**Exp 2**	44.22 ± 0.61	28.41 ± 0.54
**Exp 3**	37.71 ± 0.96	37.40 ± 0.57
**Exp 4**	49.71 ± 0.99	29.12 ± 1.10
**Exp 5**	50.09 ± 1.29	34.27 ± 1.61
**Exp 6**	38.60 ± 0.72	38.32 ± 2.07
**Exp 7**	43.89 ± 1.99	30.39 ± 0.37
**Exp 8**	51.39 ± 0.89	37.99 ± 1.16
**Exp 9**	44.21 ± 0.56	34.60 ± 2.61
**Exp 10**	40.99 ± 0.62	40.44 ± 1.84

The conditions of the experiments that showed the highest concentration of lupeol using DCM were experiment 8 (9 min, 30 mL, temperature 46°C) and using HEX were experiment 1 (3 min, 20 mL, temperature 58.65°C). In general, the experiments carried out with DCM revealed that the lupeol concentration was optimized with increasing extraction time and solvent volume. The extracts obtained with HEX showed a higher concentration of lupeol when a shorter extraction time, solvent volume, and boiling temperature were used.

The triterpenes α‐amyrin, α‐amyrin acetate, and lupeol acetate obtained in DCM extractions were also quantitatively evaluated (Table 2). Quantification was carried out through an estimated calculation using the response factor of the lupeol standard.

**TABLE 2:** Concentration of α‐amyrin, α‐amyrin acetate, lupeol, and lupeol acetate, quantitatively evaluated, present in *H. speciosa* Gomes extracts obtained by energized‐guided dispersive extraction (EDGE) using the solvent dichloromethane.

Exp.	α‐Amyrin	α‐Amyrin acetate	Lupeol	Lupeol acetate
**Exp 1**	18.30 ± 0.76	23.61 ± 3.08	40.23 ± 0.58	67.77 ± 12.32
**Exp 2**	21.18 ± 0.65	29.03 ± 1.88	44.22 ± 0.61	83.08 ± 7.21
**Exp 3**	17.31 ± 0.27	24.69 ± 0.68	37.71 ± 0.96	68.94 ± 2.79
**Exp 4**	22.89 ± 1.15	32.38 ± 0.15	49.71 ± 0.99	94.29 ± 0.72
**Exp 5**	21.55 ± 0.81	32.53 ± 1.40	50.09 ± 1.29	97.90 ± 10.27
**Exp 6**	18.34 ± 0.85	24.46 ± 3.08	38.60 ± 0.72	65.67 ± 9.53
**Exp 7**	19.15 ± 0.28	24.92 ± 2.64	43.89 ± 1.99	70.13 ± 8.94
**Exp 8**	23.18 ± 0.39	31.34 ± 3.66	51.39 ± 0.89	95.13 ± 15.06
**Exp 9**	19.55 ± 0.33	25.81 ± 2.71	44.21 ± 0.56	75.29 ± 13.99
**Exp 10**	19.65 ± 0.71	26.02 ± 1.92	40.99 ± 0.62	70.78 ± 9.45

**FIGURE 1:**
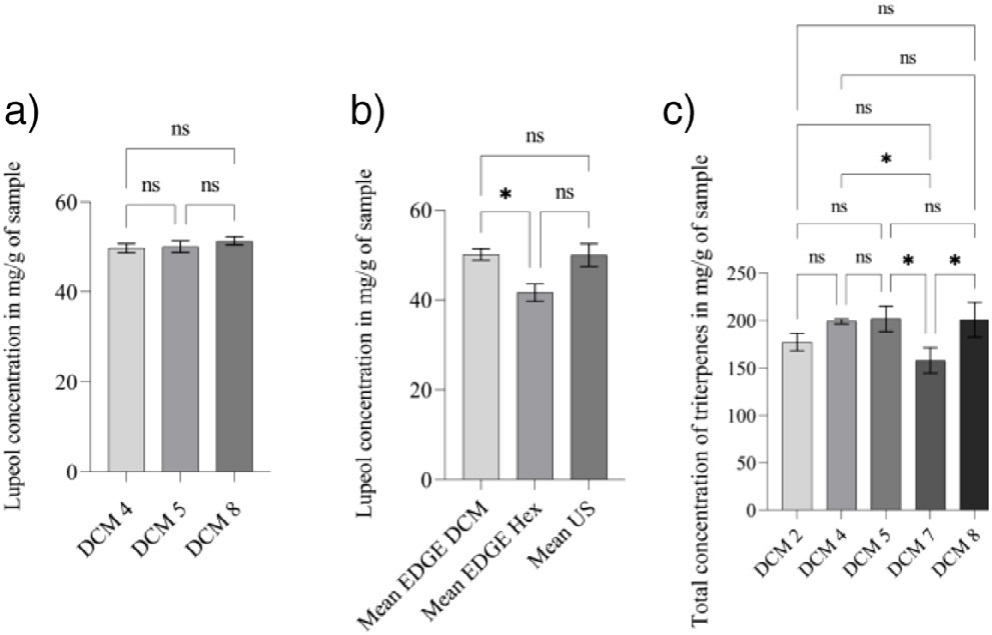
(A) Lupeol concentrations in dichloromethane (DCM) 4, DCM 5, and DCM 8 extracts via energized‐guided dispersive extraction (EDGE) exhibit no significant differences. (B) Lupeol concentrations from EDGE and ultrasound‐guided extraction. (C) Triterpene concentration, quantitatively evaluated, is present in the DCM 4, DCM 5, and DCM 8 extracts obtained by EDGE, showing no significant difference between them.

The Tukey test (Figure 1a) indicates that experiments DCM 4 (9 min, 30 mL, 34°C), DCM 5 (3 min, 20 mL, 46°C), and DCM 8 do not differ statistically in lupeol concentration (49.71, 50.09, and 51.39 mg/g, respectively). This indicates that statistically, the three experiments extracted the same amount of lupeol. The DCM 5 experiment stands out as the best configuration, as it presents shorter extraction time, smaller volume of solvent, and high efficiency in lupeol recovery. Different conditions can influence quantification, such as the interaction between different compounds.

Extracts with DCM and HEX, which presented a higher concentration of lupeol, were compared with the quantification of lupeol extracted by ultrasonic‐assisted extraction (UAE) in the best condition described in a previous study [30]. The comparison of lupeol concentrations in the three methods can be seen in Figure 1b.

From the quantitative analysis, it was observed that the experiment with the highest total amount of triterpenes was DCM 5 (Figure 1c). However, when using the Tukey test to analyze the five experiments that presented the highest concentration of triterpene, it was observed that experiments DCM 2 (9 min, 20 mL, and temperature 34°C), DCM 4, DCM 5 and DCM 8 did not show a significant difference, thus reinforcing the advantage of using the method from experiment 5, which uses a smaller volume of solvent and shorter extraction time.

It was observed that the best condition for lupeol extraction using DCM in EDGE presented a concentration of 50.09 ± 1.29 mg/g of dry sample. This value was close to that obtained using ultrasound (49.95 ± 2.54 mg/g) and higher compared to the HEX extract in EDGE (41.63 ± 1.95 mg/g).

Through Student's T‐test analysis, with a significance level of 95%, it was possible to identify that the concentration of lupeol in the DCM extract using EDGE and the ultrasonic extract did not show a significant difference. Therefore, the lupeol concentration obtained in the two extracts is statistically similar. Compared to the HEX extract using EDGE, there was a significant difference between Edge DCM and Ultrasound.

Various extraction methods exist, ranging from traditional to modern techniques. Traditional approaches, such as maceration and Soxhlet extraction, are often considered uneconomical due to their significant consumption of samples, time, energy, and particularly solvents [31]. Conversely, modern extraction techniques offer distinct advantages, including enhanced speed, improved cost‐effectiveness, greater sustainability, and a reduced environmental footprint [32].

Conversely, the UAE offers several advantages, including ease of equipment handling, enhanced safety, faster processing compared to traditional methods, and lower operational costs. Moreover, the UAE has been shown to improve the bioaccessibility of various bioactive compounds, such as vitamin C, phenolic compounds, and carotenoids [32, 33]. A drawback of the UAE, however, is the potential for damage to active constituents due to free radical formation, which can alter the extracted sample [32].

The EDGE system stands out for presenting significant advantages in relation to ultrasound extraction, such as a smaller volume of solvent (reduction of 60 mL) and shorter extraction time (reduction of 87 min). These characteristics provide greater efficiency, savings, and sustainability in the extraction process.

The result obtained was superior to those of other species, indicated in a study by Somwong and Theanphong [34] that quantified lupeol in the ethanolic extract of Derris scandens, Albizia procera, and Diospyros rhodocalyx plants by Soxhlet, obtaining a higher concentration of the compound in the Diospyros rhodocalyx extract (40.72 ± 0.40 mg/100 g). In a previous study [30], the highest concentration obtained was by ultrasonic extract of the mangaba fruit (31.21 mg/g). The difference in the lupeol concentration value obtained in the present study and that of the previous study may be related to the different period in which the samples were obtained.

### 2.3 Antioxidant Activity Test

A 2,2‐diphenyl‐1‐picrylhydrazyl (DPPH) test was carried out from 1000 to 10 µg/mL of the extract, the statistical analysis demonstrated that the concentration necessary to capture 50% of DPPH free radicals was 152.62 ± 15.08 µg/mL, making it necessary to carry out a new evaluation using a concentration range presented by statistics (300, 250, 200, 150, 100, 50, and 25 µg/mL).

In a previous study [30], *H. speciosa* fruit extracts showed greater antioxidant activity using the 2,2'‐azino‐bis(3‐ethylbenzothiazoline‐6‐sulfonic acid) method (394.5±1 µg/mL) compared to that chosen by DPPH (68.4 ± 0.3 µg/mL). Santos et al. [11], identified values in the DPPH test that varied from 125.95 to 158.67 g/g. Paula et al. [35] in DPPH analysis, it was observed that there was an increase in antioxidant capacity in freeze‐dried pulp (836.03 ± 2.05 g/g) and foam (739.58 ± 2.62 g/g).

### 2.4 Cytotoxicity Assay

**FIGURE 2:**
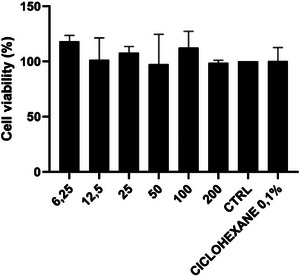
Percentage of viability of L929 fibroblasts after exposure to different concentrations of the extract, assessed by the methyl‐thiazolyl‐tetrazolium (MTT) assay.

By evaluating cytotoxicity with fibroblast cells, following the ISO 10993‐5 (2009) standard, it was possible to identify the percentage of cell viability of different concentrations of the extract under the conditions of experiment DCM 5 in triplicate. The results obtained can be seen in Figure 2.

All six tested concentrations showed a high percentage of cell viability, above 100%, with medium absorbance similar to the control. This outcome confirms the extract did not present cytotoxicity in fibroblast cells, indicating its potential for subsequent biological investigations. According to ISO 10993‐5 (2009), cell viability above 80% indicates no cytotoxicity, 60% to 80% as mild cytotoxicity, 40% to 60% as moderate cytotoxicity, and below 40% as severe cytotoxicity.

The study by Beserra et al. [36] analyzed the in vitro effect of lupeol and observed, through the methyl‐thiazolyl‐tetrazolium (MTT) test with keratinocyte or fibroblast cells, that lupeol at concentrations of 10 µg/mL and 20 µg/mL inhibited the specification of keratinocytes after 24 h of treatment in 53% and 64%, with a concentration of 1 µg/mL increasing the fibroblast barrier by 12%, while the highest concentration (20 µg/mL) inhibited the cellular barrier in relation to the control (19%). It was indicated that lupeol did not affect cell viability in keratinocytes; however, it demonstrated cytotoxicity for fibroblasts when used at a concentration of 20 µg/mL.

While the current study successfully established the non‐cytotoxic nature of the extract, future research should focus on elucidating their cellular and molecular interactions of the compounds within the extract that contribute to cell health.

### 2.5 Irritation Assay Hen's Egg Test‐Chorioallantoic Membrane

**FIGURE 3:**
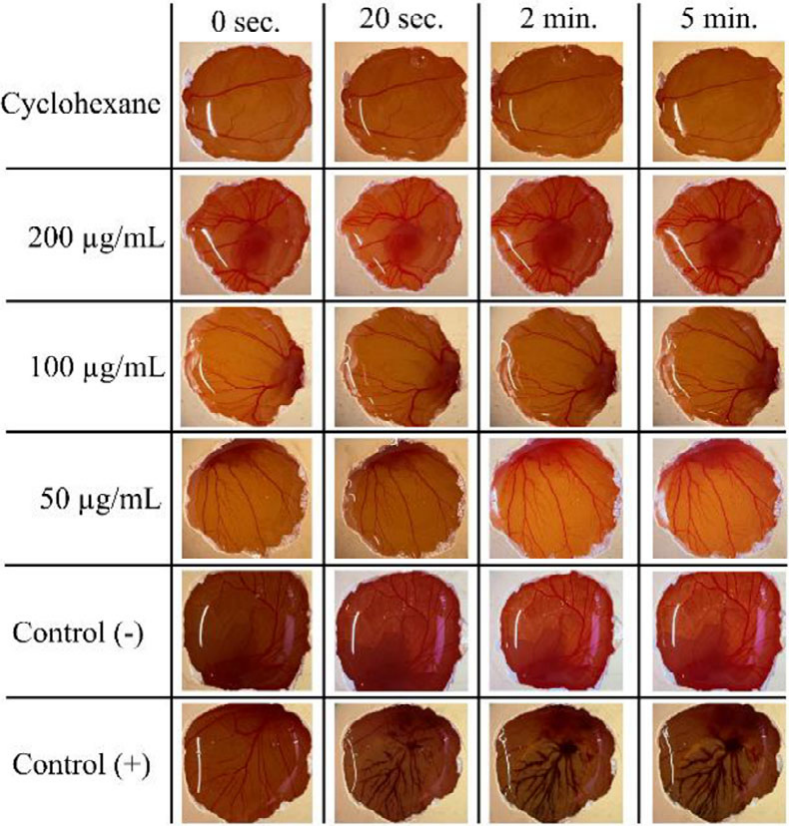
Assessment of irritation of the chorioallantoic membrane (CAM) after application of the solvent cyclohexane, *H. speciosa* extract in different concentrations (50–200 µg/mL), control (−) 0.9% sodium chloride solution, and control (+) sodium hydroxide, during a period of 5 min.

After applying *H. speciosa* extracts to the chorioallantoic membrane, the images obtained were analyzed to determine the potential for irritation in the membrane, based on the study by Luepke [37]. Figure 3 shows the assessment of irritation of the chorioallantoic membrane using extracts from *H. speciosa* under the conditions of experiment DCM 5.

From Figure 3, it is possible to observe that the extracts, across all evaluated concentrations, demonstrated no signs of irritation on the CAM membrane. The absence of any observed vascular events, such as hemorrhage, lysis, or coagulation, throughout the study period strongly supports the classification of these extracts as non‐irritating under the conditions of this assay [37]. This is a crucial finding, particularly if these extracts are intended for applications requiring direct contact with biological tissues, such as in cosmetic or dermatological formulations, where safety and mildness are paramount.

These results align with previous research on related natural compounds. In a study by Pârvănescu et al. [38], the physicochemical characterization and pharmacotoxicity of betulin and lupeol oleogel formulations, aimed at topical administration against skin lesions, were analyzed. It was recorded that, at higher concentrations, the oleogel with lupeol did not register changes in the blood vessels, nor did it generate irritation in the egg membrane.

This finding lends further support to the potential for natural products, and specifically components like those potentially present in our extracts, to exhibit favorable safety profiles regarding irritation. While the CAM assay provides a valuable initial screen for irritation potential, future studies will aim to further corroborate these findings through in vivo models to assess dermal irritation in a more physiologically relevant context.

### 2.6 Neutrophil Test

**FIGURE 4:**
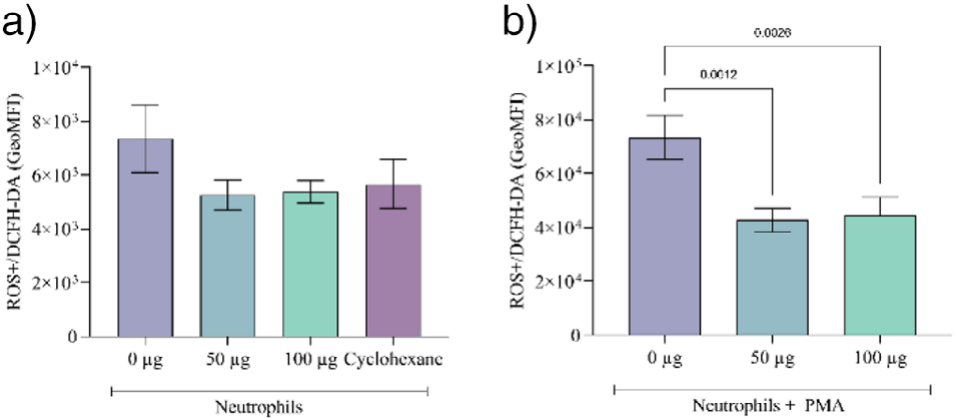
(A) Reactive oxygen species (ROS) produced in neutrophils not stimulated with Forbol 12‐myristate‐13‐acetate (PMA), where the 0 µg bar represents neutrophils not treated with extract and the Cyclohexane bar represents neutrophils treated with the extract diluting solvent. (B) ROS produced in neutrophils stimulated with PMA.

Figure 4 displays the reactive oxygen species (ROS) production in neutrophils, both under basal conditions and following Forbol 12‐myristate‐13‐acetate (PMA) stimulation. The results obtained are presented as the geometric mean (GeoMFI) of the fluorescence intensity of the 2′,7′‐dichlorofluorescin diacetate (DCFH) probe, reading color, proportionally equivalent to the amount of ROS produced in the neutrophil.

As depicted in Figure 4, no significant difference was observed in the ROS buffering capacity between the extract and the cyclohexane solvent. Neutrophils demonstrated a high production of ROS, which was significantly reduced by both concentrations of the extract.

The observed reduction in neutrophil‐derived ROS by the extract aligns with previous research on the antioxidant properties of plant species, particularly those containing compounds like lupeol. Lupeol has been implicated in preventing ROS generation and accumulation through mechanisms involving the regulation of signaling pathways such as NF‐κB [23, 39]. These findings imply a role for lupeol in the antioxidant effects observed in our study. Ribeiro et al. [40] evaluated the hepatoprotective effect of Brazilian species, among which *H. speciosa* was evaluated, where it was observed that the extract reduced ROS levels, being similar to the untreated control group and silymarin group, traditionally used to treat hepatic conditions. The authors indicate that the lower level of ROS may have occurred due to the presence of antioxidant compounds such as rutin and chlorogenic acid.

## 3 Conclusions

The EDGE system proved to be an efficient extraction method for *H. speciosa* Gomes, yielding an extract rich in triterpenes with reduced solvent consumption and a shorter extraction time. The extract obtained using the DCM extracting solvent showed a high concentration of lupeol, lupeol acetate, α‐amyrin, and α‐amyrin acetate, being higher than those obtained with HEX.

Beyond its favorable chemical profile, the DCM extract exhibited promising biological activities. It demonstrated antioxidant activity in the DPPH assay. Furthermore, the extract exhibited no cytotoxicity in the MTT assay or irritability in the hen's egg test‐chorioallantoic membrane (HET‐CAM) test, and notably, it produced a significant reduction in ROS in neutrophils at the tested concentrations.

Therefore, the use of EDGE in the extraction of the fruit of *H. speciosa* Gomes can be considered promising for future tests, and the results obtained indicate that the extract has potential phytotherapeutic applications.

## 4 Experimental

### 4.1 Sample Preparation

Mangaba fruits (*H. speciosa* Gomes) used in this study were collected on a private site (Latitude –10.996193, Longitude – 37.094002) during the month of March 2021. The use of the species for this project was registered in the Sistema Nacional de Gestão do Patrimônio Genético e do Conhecimento Tradicional Associado (SisGen) with registration number A3F5C2D.

The collected fruits (peel and pulp) were sanitized and cut into small cubes, subsequently passed through a freeze‐drying process (Liotop), then crushed with a mixer (Philips) and separated with an analytical sieve (Bertel) according to the granulometry, being used for extraction those with a granulometry of 32 mesh.

For extractions, the solvents DCM, HEX (Química Moderna) of analytical grade, Q‐Disc filters of type S1 (CEM), and a 40 mL Q‐Cup container (CEM) were used. For chromatographic analyses, the lupeol standard (Sigma‐Aldrich) and triacontane standard (Sigma‐Aldrich) were used. Helium gas (99.999%) was used in the GC/qMS analyses, purchased from White Martins (Aracaju, SE).

For testing phenolic compounds and flavonoids, Milli‐Q water, gallic acid (Perfyl tech), Folin‐Ciocalteau reagent (ACS Científica), sodium carbonate (ACS Científica), sodium nitrate (Sigma‐Aldrich), and sodium hydroxide (Synth) were used. For cytotoxicity tests, Dulbecco's Modified Eagle Medium (DMEM) (Sigma‐Aldrich) was used, containing NaHCO_3_, ampicillin, streptomycin, and supplemented with 10% fetal bovine serum (FBS), MTT (Sigma‐Aldrich), and cyclohexane P.A. (Synth).

### 4.2 Factorial Planning to Optimize Triterpene Extraction Using EDGE

With the aim of reducing solvent consumption and optimizing extraction efficiency, a full experimental design (2^3^) was used (Table 3), adapted from Tasfiyati et al. [27], using 2 g of the sample for extraction. The extract yield and triterpene concentration were considered as dependent variables. From the extracts obtained, solutions were prepared in DCM, subsequently diluted in working solutions, for analysis by gas chromatography.

**TABLE 3:** Description of the experimental design (2^3^) with central point replicated twice for extraction by energized‐guided dispersive extraction (EDGE).

Experiment	Independent variables						
	x1: Time		x2: Solvent volume		x3: Temperature		
	Code	Value (min)	Code	Value (mL)	Code	Value DCM (°C)	Value Hex (°C)
Exp 1	−1	3	−1	20	−1	34	59
Exp 2	1	9	−1	20	−1	34	59
Exp 3	−1	3	1	30	−1	34	59
Exp 4	1	9	1	30	−1	34	59
Exp 5	−1	3	−1	20	1	46	79
Exp 6	1	9	−1	20	1	46	79
Exp 7	−1	3	1	30	1	46	79
Exp 8	1	9	1	30	1	46	79
Exp 9	0	6	0	25	0	39,6	69
Exp 10	0	6	0	25	0	39,6	69

### 4.3 Ultrasound Extraction

Unique ultrasound equipment (Model USC‐1400A), with an ultrasonic frequency of 40 kHz and ultrasonic power of 135 W without heating, was utilized. After obtaining the extract, filtration was carried out with qualitative filter paper with 3 µm (Nalgon) porosity, and the solvent was evaporated in a fume hood at room temperature. The conditions for extraction were defined based on a previous study [30], where the extraction conditions utilized were: 3 g of sample, 20 mL of ethanol, 60 mL of DCM, 90 min of extraction, and a temperature of 30°C.

### 4.4 GC Analysis

To analyze the samples, a GC/qMS, model GC/qMS‐QP 2010 Ultra, from Shimadzu (Japan) was used. The capillary column used was the ZB‐5 with dimensions of 30 m in length, 0.25 mm in internal diameter, and 0.25 µm in stationary phase thickness. Temperature programming started at 40°C, held for 1 min, heated at a rate of 3°C/min, up to 300°C, which was held for 20 min. The injector temperatures were 280°C, the interface and the ion source were maintained at 300°C, the electronic impact ionization energy used was 70 eV, and the mass spectrum was scanned between 45 and 450 *m/z*. The carrier gas used was helium (He) with a flow of 0.75 mL/min. 1 µL of a 5000 mg/L solution was injected with a split ratio of 1:15.

Tentative identification of compounds using GC/qMS was performed by comparing experimentally obtained mass spectra with mass fragmentation spectra from the NIST 14 library. Retention rates were also calculated according to the equation by Van Den Dool and Kratz (1963) [41], being compared with the rates from the NIST online library available on the website http://webbook.nist.gov/chemistry/, considering the maximum difference of 10 units.

The quantification of total triterpenes was carried out in triplicate using the response factor of the lupeol standard and the internal standard triacontane (C_30_H_62_). To construct the analytical curve, GC/qMS was used in single ion monitoring mode, where ion 57 was chosen for the internal standard and ion 218 for lupeol.

For the analysis of the extracts, solutions of 1000 µg of each extract were prepared in DCM, with the addition of 10 µg of internal standard, and the analyses were carried out in triplicate.

### 4.5 Antioxidant Activity Tests

For the analysis of antioxidant activity, the DPPH method was used, where different concentrations of extracts in methanol (10–1000 µg/mL) were used. For the DPPH test, 500 µL of each extract was used, and 2 mL of a 0.1 m mol/L DPPH solution, previously prepared and incubated in the dark for 30 min, was added. The percentage of inhibition was calculated by the following formula:

$$I\%\;=\frac{A0-\mathrm{AI}}{A0}\;\times \;100$$

where A0 is the absorbance of the control and AI is the absorbance of the samples. The absorbance was read at 510 nm in a UV‐Visible spectrophotometer (Epoch 2; BioTek) using 300 µL of the final solution in a 96‐well propylene glycol plate.

### 4.6 Cytotoxicity Test (MTT)

For this test, fibroblasts of the L929 lineage were commonly seeded in 96‐well culture plates (1 × 10⁴ cells/well) and cultivated in DMEM (Sigma‐Aldrich), containing NaHCO_3_ (1.2 g/L), ampicillin (0.025 g/L), streptomycin (0.1 g/L), and supplemented with 10% FBS. The cells were subjected to fixed volume of 200 µg/mL with different doses of the extract (6.25, 12.5, 25, 50, 100, and 200 µg/mL), diluted in 0.1% cyclohexane in all extract concentrations, for 24 h, in incubator at 37°C and 5% CO_2_. The negative control was the cells in culture medium present in the plates without treatment, and the positive control was performed with the plate without cells and without extract. Cell viability was assessed by the colorimetric method using MTT. A 0.025 g solution diluted in 50 mL of PBS was placed in contact with the cells, which were then incubated at 37°C for 3 h. After removing the MTT, dimethyl sulfoxide was placed for 10 min to solubilize the tetrazole salt crystals, and then the optical density was read in an automated plate reader (enzyme‐linked immunosorbent assay) at a wavelength of 570 nm. The tests were performed in four triplicate and then normalized according to the following equation:

$$\%\;\mathrm{Cell}\;\mathrm{viability}\;=\frac{\mathrm{Abs}\;\left(\mathrm{treated}\;\mathrm{cells}\right)-\mathrm{Abs}\left(\mathrm{blank}\right)}{\mathrm{Abs}\;\left(\mathrm{positive}\;\mathrm{control}\right)-\mathrm{Abs}\;\left(\mathrm{blank}\right)}\;\times \;100\%$$

### 4.7 Irritation Assay With HET‐CAM

The toxicity test was carried out according to the method described by Barros et al. [42]. The extracts were evaporated in the fume hood, and then the dried extract was resuspended in Cyclohexane. Different concentrations of extracts (50, 100, and 200 µg/mL) were used to perform the test.

For this test, fertilized chicken eggs were incubated at 37.5°C for a period of 8 days. On the eighth day of incubation, the eggshell around the air chamber was removed, exposing the outer membrane. This membrane was hydrated with 0.9% saline solution. The eggshell membrane was removed to expose the chorioallantoic membrane. The different concentrations of the extract were deposited in a fixed volume of 300 µL on the chorioallantoic membrane and, after 5 min of exposure, irritation symptoms were analyzed, such as lysis, hemorrhage, or coagulation. A positive control (300 µL of 1 mol/L NaOH solution), a negative control (300 µL of 0.9% NaCl solution), and the solvent cyclohexane were also evaluated.

### 4.8 Anti‐inflammatory Activity Tests With human Neutrophils

Neutrophils were collected from 10 healthy donors and were treated with PMA and ROS. Measurement was performed using a DCFH probe as described by Liu et al. [43]. These neutrophils were obtained from the peripheral blood of healthy donors by density difference using the Histopaque‐1077 reagent through centrifugation and subsequent breaking of the red blood cells. When separated, the neutrophils were washed with saline solution (0.9% saline) and finally plated with culture medium suitable for mammalian cells (RMPI 1640 with antibiotics). Distributed into the wells of the plate, at a concentration of 1 × 10^6^ neutrophils, each set of cells was stimulated according to the needs of the experiment, and extract concentrations of 50 µg and 100 µg were used.

The stimulus time for ROS production and treatment with extracts was 30 min. After stimulation and treatment, the cells were washed and incubated with the fluorescent probe that estimates the amount of ROS. After incubation, the cells were analyzed by flow cytometry, model a BD FACSCanto II, where 30 thousand cells were read.

## Author Contributions

**Sérgio Prado Leite**: conceptualization, formal analysis, methodology investigation, writing – original draft. **Michel Rubens dos Reis Souza**: formal analysis, methodology investigation. **Adilson Allef Moraes Santana**: Formal analysis, Methodology investigation. **Monalisa Martins Montalvão**: formal analysis, methodology investigation. **Camilla Natália Oliveira Santos**: formal analysis, methodology investigation, **Lucas Sousa Magalhães**: formal analysis, methodology investigation, **Cristiane Bani Correa**: supervision, resources. **Tatiana Rodrigues de Moura**: supervision, resources. **Sona Jain**: supervision, writing – review and editing. **Thiago Rodrigues Bjerk**: supervision, writing – review and editing.

## Conflicts of Interest

The authors declare no conflicts of interest.

## Supporting information




**Supporting File 1**: cbdv70232‐sup‐0001‐SuppMat.docx

## Data Availability

Data will be made available on request. The chromatograms are available in the Supporting Information of this article.

## References

[1] N. Narain , F. R. M. França , and M. T. S. L. Neta , “Mangaba—Hancornia speciosa,” in Exotic Fruits Reference Guide, eds. S. Rodrigues , E. de Oliveira Silva , and E. S. de Brito (Elsevier, 2018), 305–318.

[2] J. F. Da Silva Júnior , A. V. C. D. S. Muniz , A. D. S. Lédo , et al., Descriptors for Mangaba (Hancornia speciosa Gomes) (Bioversity International and Brazilian Agriculture Research Corporation, 2018).

[3] J. de Jesus Costa , S. S. S. Sardeiro , F. de Assis Mendonça , and R. Melo e Souza , “ *Hancornia speciosa* Gomes Colonization in Restinga Environments in Tropical Climate Areas,” RA'E GA—O Espaco Geografico em Analise 49 (2020): 164–180.

[4] R. D. Vieira Neto , F. L. D. Cintra , A. da Silva Ledo , et al., “Sistema de produção de mangaba para os tabuleiros costeiros e baixada litorânea,” Embrapa Tabuleiros Costeiros Sistemas De Produção 02 (2002): 1–22.

[5] F. de Oliveira Yamashita , M. Torres‐Rêgo , J. A. dos Santos Gomes , et al., “Mangaba (*Hancornia speciosa* Gomes) Fruit Juice Decreases Acute Pulmonary Edema Induced by *Tityus serrulatus* Venom: Potential Application for Auxiliary Treatment of Scorpion Stings,” Toxicon 179 (2020): 42–52. 32174508 10.1016/j.toxicon.2020.02.025

[6] G. S. Pegorin , M. N. Leite , M. Antoniassi , et al., “Physico‐Chemical Characterization and Tissue Healing Changes by *Hancornia speciosa* Gomes Latex Biomembrane,” Journal of Biomedical Materials Research. Part B, Applied Biomaterials 109 (2021): 938–948. 33241610 10.1002/jbm.b.34758

[7] P. L. D'abadia , A. F. Costa , M. T. Firmino , et al., “The Role of Enzymes in the Angiogenic Activity of *Hancornia speciosa* Latex,” Bioscience Journal 38 (2022): e38086, 10.14393/BJ-v38n0a2022-61092.

[8] L. N. Moreira , C. Feltrin , J. E. Gonçalves , et al., “Determination of l‐(+)‐bornesitol, the Hypotensive Constituent of *Hancornia speciosa*, in Rat Plasma by LC‐MS/MS and Its Application on a Pharmacokinetic Study,” Biomedicine and Pharmacotherapy 132 (2020): 110900, 10.1016/j.biopha.2020.110900. 33113433

[9] A. B. D. Pereira , J. H. de S. Gomes , et al., “Definition of Chemical Markers for *Hancornia speciosa* Gomes by Chemometric Analysis Based on the Chemical Composition of Extracts, Their Vasorelaxant Effect and α‐Glucosidase Inhibition,” Journal of Ethnopharmacology 299 (2022): 115692, 10.1016/j.jep.2022.115692. 36084818

[10] F. L. C. Almeida , E. N. A. de Oliveira , E. C. Almeida , et al., “Mangaba (*Hancornia speciosa* Gomes) Beverage as an Alternative Wine,” Journal of Food Processing and Preservation 45 (2021), 10.1111/jfpp.15779.

[11] P. S. Santos , L. D. S. Freitas , E. N. Muniz , J. G. S. Santana , and A. V. C. Da Silva , “Phytochemical and Antioxidant Composition in Accessions of the Mangaba Active Germplasm Bank,” Revista Caatinga 34 (2021): 228–235.

[12] M. C. de Medeiros Bezerra Jácome , C. E. de Araújo Padilha , M. R. do Nascimento Arrais , A. L. O. de Sá Leitão , F. C. de Sousa Júnior , and E. S. Dos Santos , “Valorization of Mangaba Residue (*Hancornia speciosa* Gomes) for Polygalacturonase Production From Aspergillus niger IOC 4003 and Fabrication of Active Chitosan Films,” Biomass Conversion and Biorefinery 12 (2020): 4069–4080.

[13] J. F. Panontin , M. K. D. Rambo , V. Isaac , C. S. Seibert , and E. Scapin , “New Antioxidant Lauryl‐free Herbal Shampoo Formulation With a Brazilian Plant Extract,” Brazilian Journal of Biology 82 (2022): e264677, 10.1590/1519-6984.264677. 36287404

[14] R. S. Santos , A. B. Chaves‐Filho , L. A. S. Silva , et al., “Bioactive Compounds and Hepatoprotective Effect of *Hancornia speciosa* Gomes Fruit Juice on Acetaminophen‐induced Hepatotoxicity In Vivo,” Natural Product Research 36 (2022): 2565–2569. 33749461 10.1080/14786419.2021.1902324

[15] L. S. Neto , R. Q. Moraes‐Souza , T. S. Soares , et al., “A Treatment With a Boiled Aqueous Extract of *Hancornia speciosa* Gomes Leaves Improves the Metabolic Status of Streptozotocin‐induced Diabetic Rats,” BMC Complementary Medicine and Therapies 20 (2020): 114, 10.1186/s12906-020-02919-2. 32303220 PMC7164147

[16] R. Tomazi , Â. C. Figueira , A. M. Ferreira , et al., “Hypoglycemic Activity of Aqueous Extract of Latex From *Hancornia speciosa* Gomes: A Study in Zebrafish and In Silico,” Pharmaceuticals 14 (2021): 856, 10.3390/ph14090856. 34577555 PMC8472165

[17] S. A. Holanda Pinto , L. M. S. Pinto , G. M. A. Cunha , M. H. Chaves , F. A. Santos , and V. S. Rao , “Anti‐inflammatory Effect of α, β‐Amyrin, a Pentacyclic Triterpene From *Protium heptaphyllum* in Rat Model of Acute Periodontitis,” Inflammopharmacology 16 (2008): 48–52. 18046512 10.1007/s10787-007-1609-x

[18] D. L. Lucetti , E. C. P. Lucetti , M. A. M. Bandeira , et al., “Anti‐inflammatory Effects and Possible Mechanism of Action of Lupeol Acetate Isolated From *Himatanthus drasticus* (Mart.) Plumel,” Journal of Inflammation 7 (2010): 60, 10.1186/1476-9255-7-60. 21167055 PMC3019217

[19] H. R. Siddique and M. Saleem , “Beneficial Health Effects of Lupeol Triterpene: A Review of Preclinical Studies,” Life Sciences 88 (2011): 285–293. 21118697 10.1016/j.lfs.2010.11.020

[20] L. H. Vázquez , J. Palazon , and A. Navarro‐Ocaña , “Pentacyclic Triterpenes α,β‐Amyrins: A Review of Sources and Biological Activities,” in Phytochemicals‐ *A Global Perspective of Their Role in Nutrition and Health*, ed. V. Rao (IntechOpen, 2012), 487–502.

[21] F. P. Beserra , A. J. Vieira , L. F. S. Gushiken , et al., “Lupeol, a Dietary Triterpene, Enhances Wound Healing in Streptozotocin‐Induced Hyperglycemic Rats With Modulatory Effects on Inflammation, Oxidative Stress, and Angiogenesis,” Oxidative Medicine and Cellular Longevity 2019 (2019): 3182627, 10.1155/2019/3182627. 31210838 PMC6532325

[22] A. O. Nogueira , Y. I. S. Oliveira , B. L. Adjafre , M. E. A. de Moraes , and G. F. Aragão , “Pharmacological Effects of the Isomeric Mixture of Alpha and Beta Amyrin From *Protium heptaphyllum*: A Literature Review,” Fundamental and Clinical Pharmacology 33 (2019): 4–12. 30003594 10.1111/fcp.12402

[23] K. Liu , X. Zhang , L. Xie , et al., “Lupeol and Its Derivatives as Anticancer and Anti‐inflammatory Agents: Molecular Mechanisms and Therapeutic Efficacy,” Pharmacological Research 164 (2021): 105373, 10.1016/j.phrs.2020.105373. 33316380

[24] A. D. Kinross , K. J. Hageman , W. J. Doucette , and A. L. Foster , “Comparison of Accelerated Solvent Extraction (ASE) and Energized Dispersive Guided Extraction (EDGE) for the Analysis of Pesticides in Leaves,” Journal of Chromatography A 1627 (2020): 461414, 10.1016/j.chroma.2020.461414. 32823112

[25] R. Wibisono , J. Zhang , Z. Saleh , D. E. Stevenson , and N. I. Joyce , “Optimisation of Accelerated Solvent Extraction for Screening of the Health Benefits of Plant Food Materials,” Scientific Research 01 (2009): 220–230.

[26] H. Sun , X. Ge , Y. Lv , and A. Wang , “Application of Accelerated Solvent Extraction in the Analysis of Organic Contaminants, Bioactive and Nutritional Compounds in Food and Feed,” Journal of Chromatography A 1237 (2012): 1–23. 22465684 10.1016/j.chroma.2012.03.003

[27] A. N. Tasfiyati , L. D. Antika , R. T. Dewi , A. W. Septama , A. Sabarudin , and T. Ernawati , “An Experimental Design Approach for the Optimization of Scopoletin Extraction From *Morinda citrifolia* L. Using Accelerated Solvent Extraction,” Talanta 238 (2022): 123010, 10.1016/j.talanta.2021.123010. 34857344

[28] P. N. A. dos Santos , N. M. Conrado , T. M. Neubauer , A. L. dos Santos , L. C. Krause , and E. B. Caramão , “Optimization of Energized Dispersive Guided Extraction (EDGE) of Antioxidants From *Eugenia uniflora* L. (Pitanga) Leaves Using Response Surface Methodology,” Microchemical Journal 187 (2023): 108411, 10.1016/j.microc.2023.108411.

[29] M. R. R. Souza , E. Santos , A. S. Moraes , et al., “Green Extraction of Commiphora leptophloeos Mart.—J. B. Gillett Aiming to Increase the Content of Hinokinin, an “Emerging Bioactive”,” Sustainable Chemistry and Pharmacy 33 (2023): 101128, 10.1016/j.scp.2023.101128.

[30] S. P. Leite , T. B. Adami , T. R. Bjerk , et al., “Ultrasonic Assisted Extraction of Bioactive Compounds From Different Parts of *Hancornia speciosa* Gomes,” Journal of Medicinal Plants Research 14 (2020): 300–308.

[31] G. Cravotto , A. Binello , and L. Orio , “Green Extraction Techniques: For High‐quality Natural Products,” Agro Food Industry Hi Tech 22 (2011): 57–59.

[32] K. Ameer , H. M. Shahbaz , and J. H. Kwon , “Green Extraction Methods for Polyphenols From Plant Matrices and Their Byproducts: A Review,” Comprehensive Reviews in Food Science And Food Safety 16 (2017): 295–315. 33371540 10.1111/1541-4337.12253

[33] M. Hadidi , A. Ibarz , and J. Pagan , “Optimisation and Kinetic Study of the Ultrasonic‐assisted Extraction of Total Saponins From Alfalfa (*Medicago sativa*) and Its Bioaccessibility Using the Response Surface Methodology,” Food Chemistry 309 (2020): 125786, 10.1016/j.foodchem.2019.125786. 31704078

[34] P. Somwong and O. Theanphong , “Quantitative Analysis of Triterpene Lupeol and Anti‐inflammatory Potential of the Extracts of Traditional Pain‐relieving Medicinal Plants *Derris scandens*, *Albizia procera*, and *Diospyros rhodocalyx* ,” Journal of Advanced Pharmaceutical Technology & Research 12 (2021): 147–151. 34159145 10.4103/japtr.JAPTR_13_21 PMC8177155

[35] L. C. Paula , F. A. Silva , E. P. Silva , E. R. Asquieri , and C. Damiani , “Influence of Preservation Methods on the Bioactivity of Mangaba (*Hancornia speciosa* Gomes) From the Brazilian Savannah,” Food Science and Technology 39 (2019): 403–409.

[36] F. P. Beserra , M. Xue , G. L. De Azevedo Maia , A. L. Rozza , C. H. Pellizzon , and C. J. Jackson , “Lupeol, a Pentacyclic Triterpene, Promotes Migration, Wound Closure, and Contractile Effect In Vitro: Possible Involvement of PI3K/Akt and p38/ERK/MAPK Pathways,” Molecules 23 (2018): 2819, 10.3390/molecules23112819. 30380745 PMC6278408

[37] N. P. Luepke , “Hen's Egg Chorioallantoic Membrane Test for Irritation Potential,” Food and Chemical Toxicology 23 (1985): 287–291. 4040077 10.1016/0278-6915(85)90030-4

[38] E. Szliszka , Z. P. Czuba , M. Domino , B. Mazur , G. Zydowicz , and W. Krol , “Ethanolic Extract of Propolis (EEP) Enhances the Apoptosis‐inducing Potential of TRAIL in Cancer Cells,” Molecules 14 (2009): 738–754, 10.3390/molecules. 19223822 PMC6254026

[39] F. S. Tsai , L. W. Lin , and C. R. Wu , “Lupeol and Its Role in Chronic Diseases,” Advances in Experimental Medicine and Biology 929 (2016): 145–175. 27771924 10.1007/978-3-319-41342-6_7

[40] G. dos Santos Ribeiro , D. H. N. Martins , J. V. D. Gomes , et al., “Hepatoprotective Effects of Four Brazilian Savanna Species on Acetaminophen‐Induced Hepatotoxicity in HepG2 Cells,” Plants 12 (2023): 3393, 10.3390/plants12193393. 37836133 PMC10574628

[41] H. Van Den Dool and P. D. Kratz , “A Generalization of the Retention Index System Including Linear Temperature Programmed Gas—Liquid Partition Chromatography,” Journal of Chromatography A 11 (1963): 463–471. 10.1016/s0021-9673(01)80947-x 14062605

[42] L. A. B. Barros , A. L. Santos , A. S. Polidoro , et al., “Phytochemical Analysis, Antioxidant Activity and In Vitro Ocular Irritation of *Hibiscus rosa‐sinensis* L. Extracts,” International Journal of Advanced Engineering Research and Science 7 (2020): 137–149.

[43] T. Liu , F. Xiang , J. Tan , et al., “Ultrasmall Copper‐Based Nanoparticles for Reactive Oxygen Species Scavenging and Alleviation of Inflammation Related Diseases,” Nat Commun 11 (2020): 1–16. 32493916 10.1038/s41467-020-16544-7 PMC7270130

