# Supplementary material for: Extraction of Triterpene Compounds From Hancornia speciosa Gomes Fruits and Evaluation of Pharmacological Potential
Source: Chem Biodivers. 2025 Jul 14;22(11):e01034. doi: 10.1002/cbdv.202501034 (PMC12629162; doi:10.1002/cbdv.202501034)
Supplement: Supplementary file 1 — Supporting File 1: cbdv70232‐sup‐0001‐SuppMat.docx [file CBDV-22-e01034-s001.docx]

Supplementary Material

**
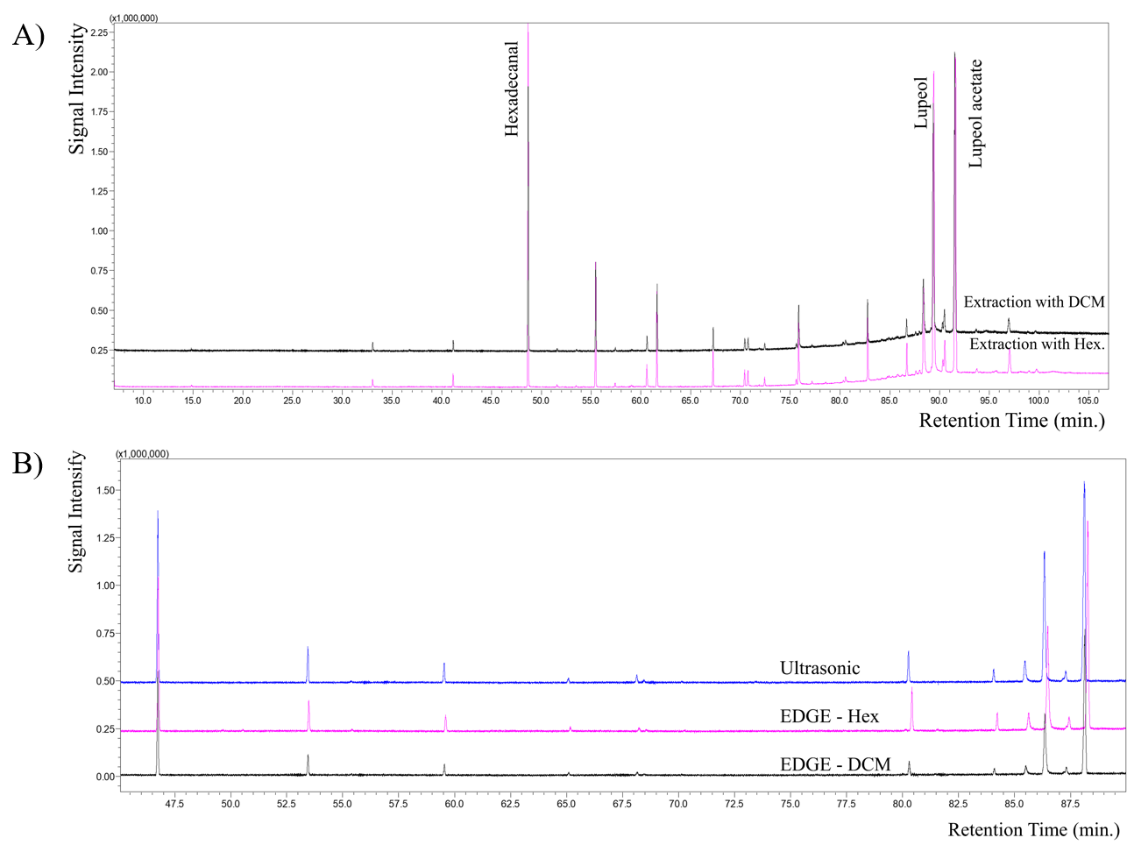
**

**Figure 1S.** A) Comparison between chromatograms (GC/qMS) for DCM and Hexane extracts from Mangabeira fruits obtained by EDGE. B) Comparison between chromatograms (GC/qMS) for extracts in DCM and Hexane obtained by EDGE and extract obtained by ultrasound.
